# FGF dependent regulation of *Zfhx1b *gene expression promotes the formation of definitive neural stem cells in the mouse anterior neurectoderm

**DOI:** 10.1186/1749-8104-5-13

**Published:** 2010-05-06

**Authors:** Lan TH Dang, Vincent Tropepe

**Affiliations:** 1 Department of Cell and Systems Biology, Centre for the Analysis of Genome Evolution and Function, University of Toronto, Toronto, ON, M5S 3G5, Canada

## Abstract

**Background:**

Mouse definitive neural stem cells (NSCs) are derived from a population of LIF-responsive primitive neural stem cells (pNSCs) within the neurectoderm, yet details on the early signaling and transcriptional mechanisms that control this lineage transition are lacking. Here we tested whether FGF and Wnt signaling pathways can regulate *Zfhx1b *expression to control early neural stem cell development.

**Results:**

By microinjecting FGF8b into the pro-amniotic cavity *ex vivo *at 7.0 days post-coitum (dpc) and culturing whole embryos, we demonstrate that neurectoderm-specific gene expression (for example, *Sox2*, *Nestin*, *Zfhx1b*) is increased, whereas Wnt3a represses neurectoderm gene expression. To determine whether FGF signaling also mediates the lineage transition from a pNSC to a NSC, 7.0-dpc embryos were microinjected with either FGF8b or inhibitors of the FGF receptor-MAP kinase signaling pathway *ex vivo*, cultured as whole embryos to approximately 8.5 dpc and assayed for clonal NSC colony formation. We show that pre-activation of FGF signaling in the anterior neurectoderm causes an increase in the number of colony forming NSCs derived later from the anterior neural plate, whereas inhibition of FGF signaling significantly reduces the number of NSC colonies. Interestingly, inhibition of FGF signaling causes the persistence of LIF-responsive pNSCs within the anterior neural plate and over-expression of *Zfhx1b *in these cells is sufficient to rescue the transition from a LIF-responsive pNSC to an FGF-responsive NSC.

**Conclusion:**

Our data suggest that definitive NSC fate specification in the mouse neurectoderm is facilitated by FGF activation of *Zfhx1b*.

## Background

Mouse neural induction depends on synergistic interactions among three primitive tissues: anterior ectoderm, anterior visceral endoderm, and anterior mesendoderm derivatives of the early gastrula organizer [1]. Transforming growth factor (TGF)β/bone morphogenetic protein (BMP), Wnt and fibroblast growth factor (FGF) are three major signaling pathways known to function during these complex tissue interactions and insight into the relative contribution of each of these pathways for early development has been gleaned from targeted deletion studies. For example, the absence of *Wnt3a *results in ectopic neural tissue formation at the expense of paraxial mesoderm derivatives [2], and the loss of the TGFβ-related signaling components *Smad4 *[3], *Bmpr1a *[4] or *Nodal *[5] in the primitive ectoderm leads to precocious neural differentiation and a loss of pluripotency. Together, these data suggest that the attenuation of Wnt, BMP and Nodal signaling pathways are necessary for neural cell fate specification of the mouse anterior ectoderm, a mechanism that is broadly conserved among vertebrates [6]. The requirement for FGF signaling is less apparent. For instance, the *Fgf4 *and *Fgfr2 *null mutations are embryonic lethal during blastocyst implantation [7,8]. Furthermore, mouse embryos harboring *Fgfr1-/- *or *Fgf8-/- *mutations, which die at approximately 8.5 to 9.5 days post-coitum (dpc), indicated that FGF signaling was not required for the earliest steps in neural specification of the ectoderm, although it was necessary for normal gastrulation and neurulation [9-11]. In contrast, studies in chick [12] and *Xenopus *[13] provide evidence for an early role of FGF signaling in specifying a neural identity. Thus, it is possible that the early pleiotropic effects of FGF signaling may act to partially obscure a specific localized role during mouse neural cell fate specification.

Embryonic stem (ES) cell models of neural induction have been instrumental in complementing the mouse *in vivo *studies [14]. Indeed, Wnt and/or BMP inhibition within ES cell cultures results in a clear enhancement of neural specification and neuronal differentiation. For example, inhibition of Wnt signaling using DKK1 [15] or Sfrp2 [16] showed enhanced neural differentiation. Similarly, the BMP antagonist Noggin can enhance neural cell fate specification of ES cell cultures in serum-free and feeder-free conditions [17], whereas in the presence of a potent neural inducing feeder cell line, exogenous BMP4 can suppress neural induction [18]. These *in vitro *findings are consistent with the *in vivo *evidence linking the inhibition of BMP and Wnt signaling with mouse neural cell fate specification [19].

In contrast, there are conflicting views over whether FGF acts as a positive inducing signal or whether it plays a more permissive role during neural cell fate specification of mouse ES cells. For example, blocking FGF signaling pharmacologically or genetically under low cell density, serum-free and feeder-free conditions does not prevent ES cells from acquiring a neural cell identity after 24 hours [20]. However, many of the neural cells are specified to a 'primitive' neural state, whereby they express primarily neuroectodermal genes (for example, *Sox1*, *Nestin*), rather than mesodermal or endodermal genes, but also maintain low levels of expression of the pluripotency gene *Oct4 *[20]. The term 'primitive' reflects the fact that although these cells are specified to a neural identity, they are not yet committed (that is, definitive) [21]. On the other hand, others have shown that ES cells harboring an *Fgf4-/- *or *Erk2-/- *mutation are significantly deficient in their capacity to be induced to a definitive neural identity (Sox1+, Nestin+, Oct4-) [22]. In these experiments, however, neural induction was typically assayed over the course of several days, making it difficult to completely rule-out a permissive role for FGF signaling on proliferation of cells specified to a neural identity independently of FGF [19]. An alternative model, which would reconcile these somewhat disparate conclusions, posits that FGF signaling is required for the transition from a primitive neural identity to a definitive neural identity within the ectoderm during neural plate formation *in vivo*, or during ES cell differentiation *in vitro*, irrespective of its influence on proliferation.

Previous studies have reported that the anterior ectoderm (approximately 5.5 to 7.5 dpc) contains primitive neural stem cells (pNSCs), which when isolated in clonal *in vitro *assays display self-renewal properties, express neural genes and have the potential to generate differentiated neuronal and glial progeny [23]. pNSC-derived colonies differ from definitive neural stem cell (NSC) colonies in two main respects: they require exogenous leukemia inhibitory factor (LIF) for colony formation in primary cultures, instead of FGF; and they express relatively low levels of the pluripotency gene *Oct4*, which enables a broader differentiation potential under the influence of non-neural tissue environments [17,23]. Definitive NSCs, first evident at 8.5 dpc in the anterior neural plate, require FGF to stimulate clonal colony formation from primary dissections and are committed to a neural identity [24]. Thus, the differential growth factor requirement for colony formation serves as a useful feature to distinguish between these two stem cell populations during the course of NSC lineage ontogeny.

In the present study we tested the hypothesis that FGF signaling is required for the transition from a pNSC identity to a NSC identity by manipulating FGF signaling directly within the mouse ectoderm *ex vivo *and then assaying for the presence or absence of pNSC and NSC clonal colony-formation from stem cells isolated from the neural plate. We report that the activation of FGF signaling in the ectoderm promotes NSC development. We also describe a novel role for the zinc-finger homeobox gene *Zfhx1b *(also known as *SIP1 *or *Zeb2*) downstream of FGF signaling. *Zfhx1b *is required for normal neural tube development as well as neural crest specification [25], is a known downstream transcriptional target of FGF signaling during chick neural induction [26], and is sufficient to induce a neural cell fate in *Xenopus *ectoderm [27,28]. Interestingly, we find that ectopic expression of *Zfhx1b *in the 7.0-dpc anterior ectoderm is not only sufficient to facilitate the transition from a pNSC to NSC, but can also initiate ectopic neural plate-like tissue formation. Thus, our data suggest that definitive NSC fate specification in the mouse anterior neurectoderm is facilitated by FGF-dependent activation of *Zfhx1b*.

## Results

### Transient modulation of FGF and Wnt signaling can regulate gene expression in the mouse ectoderm

**Figure 1:**
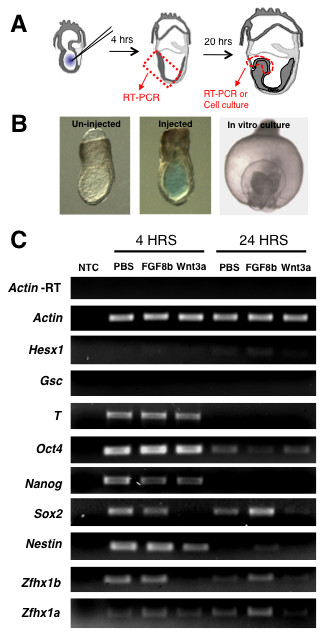
**Microinjection of FGF or Wnt3a modulates neural gene expression in the ectoderm and neural plate**. **(A) **Schematic of experimental setup. **(B) **Embryos at 7.0 dpc that were either uninjected (left) or injected with a PBS solution containing 0.05% Fast Green dye (middle) within the pro-amniotic cavity. On the far right is an anterior view (dorsal to the top) of an injected embryo after 24-hour whole embryo culture showing normal progression through gastrulation. **(C) **Semi-quantitative RT-PCR of tissue isolated at 4 hours or 24 hours post-injection and assayed for expression of neurectoderm specific genes (*Nestin*, *Sox2*, *Hesx1*, *Zfhx1b*, *Zfhx1a*), pluripontentiality genes (*Oct4 *and *Nanog*), or mesodermal genes (*Brachyury *(*T*), *Goosecoid *(*Gsc*)). *Actin *gene expression was used for relative quantification and *Actin*-RT was a control for genomic DNA contamination. Experiment was performed with two repeats on three biological samples, five embryos per sample.

To determine whether mouse ectoderm cells are competent to respond to acute modulation of FGF and Wnt signaling pathways, we used an *ex vivo *microinjection strategy (Figure 1A). Recombinant signaling proteins were microinjected into the pro-amniotic cavity of approximately 7.0-dpc mouse embryos by penetrating a glass micropipette through the primitive streak on the posterior side of the embryo and injecting a volume of approximately 200 pL close to the anterior ectoderm surface of the embryo. A glass holding micropipette placed on the extraembryonic portion was used to stabilize the embryo during injection. This optimal injection volume was determined empirically, and caused only a very slight, transient swelling of the pro-amniotic cavity upon injection, but was not sufficient to damage the embryo or compromise further *ex vivo *growth. For instance, 80-85% of all injected embryos developed normally after overnight whole embryo culture and this rate was not significantly different from that of uninjected embryos. Figure 1B shows embryos microinjected with PBS containing Fast Green dye as an indicator. The concentration of recombinant proteins was determined through dose-response experiments (data not shown) as well as from reports in the literature, and we used semi-quantitative RT-PCR or colony forming assays (see below) to evaluate the effectiveness of the concentrations used. Finally, we used 1:1 DMEM:FBS media to support further *ex vivo *growth (up to 24 hours post-treatment). We tested whether ES cell grade FBS was a suitable alternative to freshly prepared rat serum by scoring for the number of uninjected 7.0-dpc embryos that progressed through gastrulation to the head-fold stage, including visible evidence of somites, branchial arches, neural plate, alantois and optic pits as well as an amniotic cavity (Figure 1B). These features were present in embryos grown in DMEM:rat serum and DMEM:FBS at equal frequency and were indistinguishable with staged controls. Cultured embryos that did not have these morphological features after overnight culture were not further analyzed.

As mentioned above, both the *Fgf8-/- *and the *Fgfr1-/- *mutant mice display overt neural specification, but subsequent neural plate formation is severely compromised [9,11]. Thus, we reasoned that FGF signaling may not be required for the initial specification of a neural identity, but it may play a direct role in regulating the transition to a committed neural identity. Using semi-quantitative RT-PCR we observed that direct exposure of the ectoderm to FGF8b enhances and/or stabilizes neural gene expression indicated by the higher levels of *Sox2*, *Nestin*, *Zfhx1b *and *Zfhx1a *compared to the PBS-injected control embryos within 24 hours (Figure 1C). In contrast, FGF8b injection had no effect on the expression of the mesodermal genes *Goosecoid *(*Gsc*) and *Brachyury *(*T*), or the pluripotency genes *Oct4 *and *Nanog*, as these remain relatively unchanged compared to the PBS injection control (Figure 1C). We also observed a relatively small increase in the expression of *Hesx1 *in response to FGF8b at 24 hours, a gene that is expressed in the anterior neurectoderm and required for anterior neural plate development [29]. The opposite result was observed when the ectoderm was exposed to Wnt3a, which caused a decrease in neuroectoderm gene expression (*Sox2*, *Zfhx1b*) as early as 4 hours post-injection. By approximately 24 hours, Wnt3a had a strong repressive effect on most of the genes that we assayed, except for *Oct4 *(Figure 1C). Microinjection into the pro-amniotic cavity allowed for the direct exposure of ectoderm cells to exogenous recombinant proteins as opposed to bathing the embryos in growth factor-containing media, which is less sensitive for assessing the effects on neurectodermal gene expression during the relatively short developmental time window we are investigating (Additional file 1A). We also tested whether FGF8b altered the anteroposterior patterning of the neural plate by examining the expression of *Otx2 *(forebrain and midbrain), *En2 *(midbrain-hindbrain boundary) and *Gbx2 *(hindbrain). Acute exposure of the 7.0-dpc ectoderm to FGF8b did not result in any obvious changes in expression of these three genes (Additional file 1B), suggesting that the patterning of relatively broad domains within the neural plate is not significantly affected. However, more detailed analyses using markers with finer expression domains would be necessary to determine if there are more subtle alterations in anteroposterior patterning. In summary, these results indicate that FGF and Wnt signaling can acutely modulate neural cell fate specification within the anterior ectoderm *ex vivo*.

### FGF and Wnt signaling cooperatively regulate neural stem cell development within the mouse embryo

**Figure 2:**
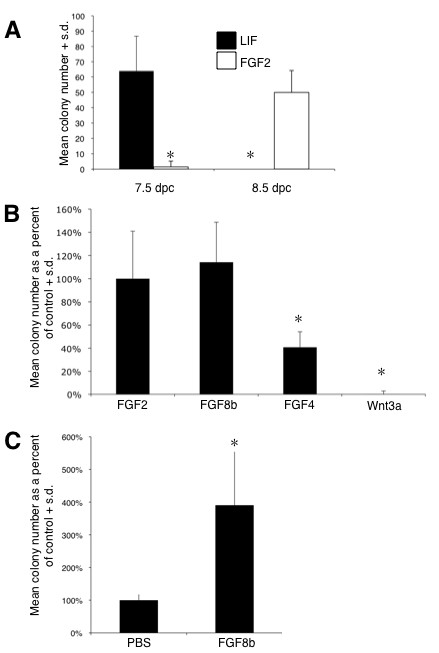
**FGF and LIF stimulate separate populations of colony forming neural stem cells at distinct times in anterior neurectoderm development**. **(A) **Clonal cell cultures derived from *ex vivo *cultured tissue dissected at either 7.5 or 8.5 dpc and maintained in serum-free media in the absence of growth factors, or in the presence of either LIF or FGF2 + heparin (H). The number of colonies derived after 14 days *in vitro *was quantified (n = 3; 4 wells/n; asterisks denote statistical significance from stage specific control, *P *< 0.05) **(B) **Quantification of FGF responsive colonies in the presence of FGF2, FGF8b or FGF4 and compared to Wnt3a (three separate experiments; n = 2 to 3 embryos for each treatment group; four replicates per culture condition per treatment in each experiment; asterisks denote statistical significance from FGF2 + H control, *P *< 0.05). **(C) **Anterior neural plate cultures derived from embryos injected with either PBS or FGF8b at 7.0 dpc and cultured until 8.5 dpc. The number of FGF responsive colonies was quantified after 14 days *in vitro *(four separate experiments; n = 2 to 3 embryos for each treatment group; four replicates per culture condition per treatment in each experiment; asterisks denote a statistical significance from PBS injection control, *P *< 0.05). S.d., standard deviation.

As described above, a current model for NSC ontogeny posits that the definitive NSC identity is preceded by a pNSC stage and that these separate populations of stem cells can be distinguished using a clonal colony-forming assay in the presence of either exogenous LIF or FGF2. Consistent with the results from previous experiments [23], we demonstrated that during *ex vivo *embryo development LIF-dependent pNSCs can be isolated from approximately 7.5 dpc, after which time they transition to an FGF-dependent NSC state (Figure 2A). As has been previously demonstrated [23], LIF responsive colonies and FGF responsive colonies can be passaged *in vitro *in the presence of FGF2, and colony cells can differentiate into neurons and glia in the presence of 5% FBS (data not shown).

Given our results that exogenous FGF8b and Wnt3a signaling could affect neural gene expression in the ectoderm (Figure 1C), we asked whether these changes in gene expression affect the transition from a pNSC to a NSC identity within the embryo by assaying for NSC colony formation. *Fgf2 *is not required for mouse neural induction, even though it does regulate neural precursor cell proliferation in the neural tube [30]. On the other hand, *Fgf8 *expression within the anterior neural ridge is sufficient to induce an anterior forebrain identity [31], suggesting that FGF8 may be the predominant signal mediating early NSC development *in vivo*. Thus, we first compared the effectiveness of FGF8b to stimulate definitive NSC clonal colony formation. In Figure 2B we show that FGF2 and FGF8b are equally effective at promoting colony formation, whereas the number of colonies observed in the presence of FGF4 was 60% less than with FGF2, and Wnt3a was incapable of facilitating colony formation (Figure 2B). Moreover, microinjection of FGF8b into the pro-amniotic cavity resulted in an approximately three-fold increase in the number of FGF2-responsive NSCs that were subsequently derived from the anterior neural plate (Figure 2C). These data suggest that modulating the levels of FGF8b on an intact ectoderm is sufficient for regulating NSC development during the formation of the anterior neural plate.

**Figure 3:**
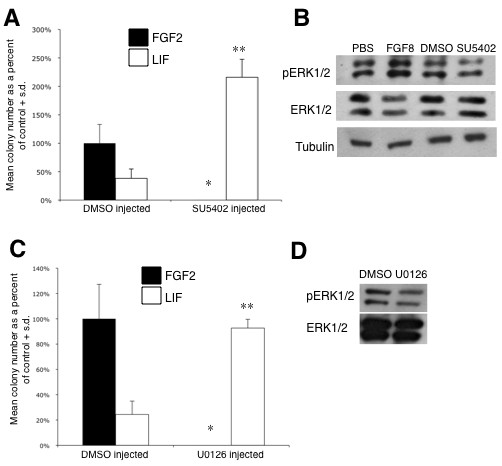
**FGF receptor and MAPK signaling inhibition maintains pNSC identity and prevents the transition to a NSC identity in the anterior ectoderm**. **(A) **Inhibition of FGF receptor using SU5402 (five separate experiments; n = 2 embryos for each treatment group; four replicates per culture condition in each experiment). **(B) **Analysis of phospho-Erk1/2 levels in the presence of FGF8b or SU5402, compared to total Erk1/2 protein levels. **(C) **Similar experiment using the MEK inhibitor, U0126 (three separate experiments; n = 2 to 3 embryos for each treatment group; four replicates per culture condition per treatment in each experiment). *Statistical significance compared to the positive control dimethyl sulfoxide (DMSO) injected, FGF2 + heparin (H) cultured, *P *< 0.05; **statistical significance compared to DMSO injected, LIF cultured, *P *< 0.05. **(D) **Phospho-Erk1/2 levels in the presence of U0126 compared to total Erk1/2 protein levels. S.d., standard deviation.

Next, using microinjection into the proamniotic cavity, we exposed the ectoderm at 7.0 dpc to the FGF receptor-specific inhibitor SU5402 [32], or the Wnt extracellular antagonist DKK1 [15], and assayed for the presence of LIF-dependent pNSCs as well as FGF-dependent NSCs within the anterior neural plate following an approximately 24 hour *ex vivo *culture. Inhibition of FGF signaling using a transient exposure of 5 μM of SU5402 in the mouse ectoderm resulted in a complete loss of FGF-responsive NSCs subsequently present in the anterior neural plate (Figure 3A). The numbers of LIF responsive pNSCs in the 7.5-dpc anterior ectoderm after exposure of SU5402 at 7.0 dpc were not significantly different from control values (Additional file 2). However, we observed a more than two-fold increase in the number of LIF responsive pNSCs that remained within the 8.5-dpc neural plate, at a time when these stem cells are barely detectable (Figure 2A). Because FGF-dependent neural induction of ES cells was previously shown to depend on mitogen-activated protein kinase (MAPK) signal transduction [22], we asked whether the MAPK pathway in ectoderm cells was modulated in response to FGF8b and SU5402. We analyzed phospho-Erk1/2 by immunoblot using ectoderm tissue dissected from injected embryos and found that phospho-Erk1/2 levels were increased in the presence of FGF8b and decreased in the presence of SU5402 (Figure 3B). A role of MAPK signaling during the transition from pNSC to NSC was further examined by microinjection of 20 μM of the MEK inhibitor U0126 [33] at 7.0 dpc. Consistent with our results using SU5402, we observed a complete loss of FGF-responsive NSCs subsequently present in the 8.5 dpc neural plate and a concomitant increase (approximately four-fold) in the number of LIF-responsive pNSC (Figure 3C), and this was correlated with a reduced level of phospho-Erk1/2 (Figure 3D). Together, these data demonstrate that MAPK-dependent FGF signaling is required for the transition to a definitive NSC identity.

**Figure 4:**
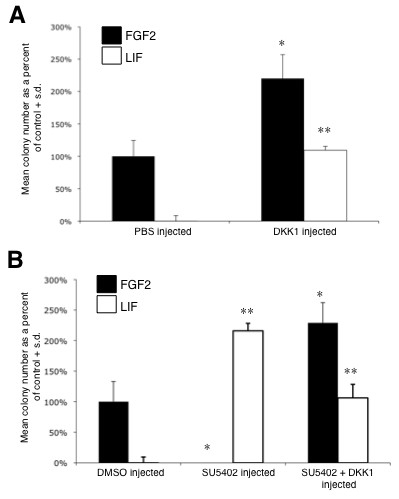
**Inhibition of Wnt signaling by DKK1 enhances the number of primitive and definitive neural stem cells in the neural plate**. **(A) **Effect of inhibition of Wnt signaling on colony formation (three separate experiments; n = 2 to 3 embryos for each treatment group; three replicates per culture condition per treatment in each experiment). **(B) **Effect of SU5402 with or without DKK1 on colony formation (four separate experiments; n = 2 to 3 embryos for each treatment group; four replicates per culture condition per treatment in each experiment). *Statistical significance compared to the positive control dimethyl sulfoxide (DMSO)/PBS injected, FGF2 + heparin (H) cultured, *P *< 0.05; **statistical significance compared to DMSO/PBS injected, LIF cultured, *P *< 0.05. S.d., standard deviation.

In contrast to the above observations, inhibition of the canonical Wnt signaling pathway at 7.0 dpc using the antagonist DKK1 showed a significant increase in both LIF-responsive pNSCs and FGF-responsive NSCs subsequently present within the 8.5-dpc neural plate (Figure 4A). There are at least two possible mechanisms to explain this observation. First, Wnt inhibition acts upstream in the lineage, enhancing neural specification in general and/or the specification of pNSCs within the ectoderm. In this model, the increase in FGF responsive NSCs would be due to the fact that they are the lineage descendents of the expanded pNSC population. Alternatively, Wnt inhibition may also influence the transition from a pNSC to a definitive NSC identity independently of FGF signaling. To distinguish between these two possibilities, we co-injected both SU5402 and DKK1. We predicted that if Wnt inhibition was primarily acting upon pNSCs, and not on NSCs, then we would observe a similar increase in the number of pNSCs that are present in the neural plate, but a concomitant loss of definitive NSCs (due to the presence of SU5402). As expected, microinjection of SU5402 alone (as a control) eliminated the presence of NSCs and enhanced the number of pNSCs that persisted to the 8.5-dpc stage (Figure 4B). However, the simultaneous inhibition of both signaling pathways at 7.0 dpc resulted in significantly higher numbers of pNSCs as well as NSCs present in the anterior neural plate at 8.5 dpc (Figure 4B). The fact that 8.5-dpc cells respond to exogenous FGF2 in the media suggests that these cells are not refractory to FGF-dependent proliferation due to lingering exposure to SU5402. We further confirmed this in embryos pre-treated with SU5402 by showing that pErk1/2 levels increased to control values in response to exogenous FGF2 after rinsing and dissecting anterior neural plate tissue (the same procedure used in preparation for the colony-forming assay) (Additional file 2B). Therefore, these results suggest that both FGF and Wnt signaling may regulate the transition from a pNSC state to a NSC state by independently converging upon similar downstream (for example, transcriptional) mechanisms.

### FGF signaling acts through Zfhx1b and is sufficient for the transition from a pNSC to a NSC state in the mouse embryo

**Figure 5:**
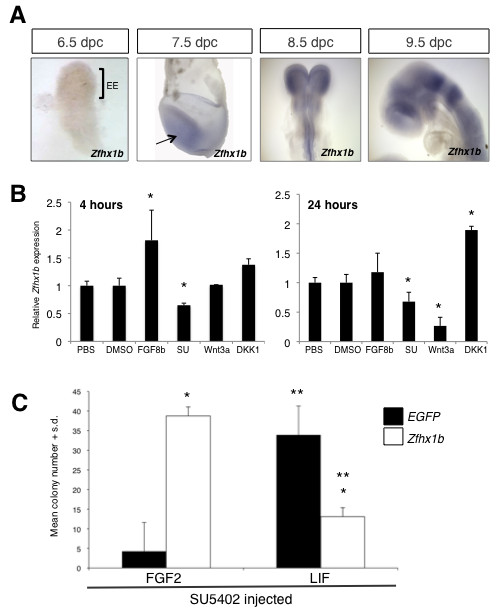
**The *Zfhx1b *gene is modulated by FGF and Wnt signaling and is sufficient to rescue FGF-responsive neural stem cell colonies in the presence of SU5402**. **(A) **Expression profile of *Zfhx1b *by whole mount *in situ *hybridization. Arrow, anterior ectoderm; EE, extraembryonic ectoderm. **(B) **Modulation of FGF and Wnt signaling alters the expression of *Zfhx1b *as shown by quantitative RT-PCR. Tissue samples prepared as in Figure 1. **(C) **Pre-treated 8.5-dpc anterior neural plate cells were transfected with an empty vector (*EGFP*) or the *Zfhx1b *over-expression construct and cultured in serum-free media for 14 days (three separate experiments; n = 2 to 3 embryos for each treatment group; two replicates per culture condition per treatment). *Statistically significant (*P *< 0.05) compared to the transfection control *EGFP *transfected in either culture conditions; ** statistical significance (*P *< 0.05) of the respective transfection in the two culture conditions, FGF2 + heparin (H) or LIF. S.d., standard deviation.

We investigated whether the FGF-dependent definitive NSC identity depends upon *Zfhx1b *expression. First, we examined its expression pattern by in situ hybridization using a gene-specific probe [34] during the onset of definitive NSC development. *Zfhx1b *is first expressed between 7.0 and 7.75 dpc in the ectoderm (Figure 5A). Consistent with previous findings [25], expression is localized to the neural plate at 8.5 dpc and neural tube germinal zone at 9.5 dpc (Figure 5A). The onset of expression correlates with the transition from a pNSC to a NSC state in the anterior ectoderm. Next, we confirmed by quantitative RT-PCR that FGF signaling could modulate endogenous levels of *Zfhx1b *in the mouse ectoderm following the microinjection of recombinant FGF8b or SU5402. Figure 5B shows that the endogenous levels of *Zfhx1b *transcript are significantly increased in the presence of FGF8b after 4 hours post-injection, which is consistent with our semi-quantitative RT-PCR data (Figure 1C), and significantly decreased in the presence of SU5402 at 4 and 24 hours post-injection (Figure 5B). Interestingly, we also found that Wnt signaling can alter *Zfhx1b *expression, but only starting at approximately 24 hours post-injection (Figure 5B). This finding is consistent with our observation of the 'rescued' FGF-dependent NSC colony formation in embryos co-injected with SU5402 and Dkk1 (Figure 4B), because it demonstrates that inhibition of the Wnt signaling pathway may independently up-regulate *Zfhx1b *gene expression, which is sufficient to promote the transition to a definitive NSC identity despite the reduced levels of FGF signaling during the transition.

In order to determine that *Zfhx1b *functions downstream of FGF signaling, and mediates the transition between a pNSC and a NSC state, we asked whether the transient over-expression of *Zfhx1b *can rescue the lack of transition of a pNSC to a NSC following the exposure of the ectoderm to the FGF receptor inhibitor SU5402. Transient transfection of the mouse *Zfhx1b *DNA construct was effective for inducing increased levels of mRNA transcript and Zfhx1bprotein in HEK293T cells (Additional file 3A), and transfection of primary low density neural plate cells post-injection resulted in approximately 75 to 80% expressing cells based on GFP reporter expression (Additional file 3B). As expected, very low numbers of FGF-responsive colonies and relatively high numbers of LIF-responsive colonies were derived from 8.5-dpc neural plate cells (from SU5402 pre-injected embryos) transfected with the *EGFP* (enhanced green fluorescent protein) control vector (Figure 5C). However, transfection of *Zfhx1b *in primary dissociated neural plate cells (from SU5402 pre-injected embryos) caused a significant increase (approximately seven-fold) in subsequent FGF-dependent colony formation and a concomitant decrease in LIF-dependent colony formation (Figure 5C). GFP expression was observed in cells within colonies, but due to the transient nature of expression the pattern was often mosaic or of varying intensity by the end of the culture period (data not shown). Thus, ectopic *Zfhx1b *is sufficient to facilitate the transition from a pNSC to a NSC when FGF signaling is blocked.

**Figure 6:**
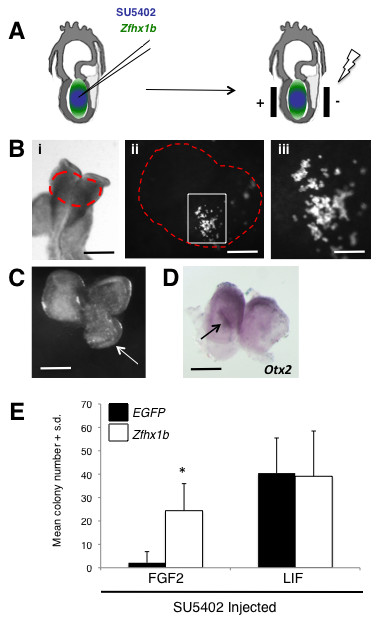
***Zfhx1b *is sufficient to induce a definitive neural stem cell identity in the anterior ectoderm**. **(A) **Schematic of the microinjection and electroporation procedures. **(B) **Electroporated embryo after 24-hour culture; the red dashed area in (i) denotes where the EGFP reporter can be visualized in cells of the anterior neural plate (scale bar: 250 μm). (ii) Magnified area of the dorsal head from (i) (scale bar: 50 μm); white box denotes region of transfected cells. (iii) Magnified area in the white box from (ii) (scale bar: 25 μm). **(C) **Over-expression of *Zfhx1b *is sufficient to induce ectopic neural plate/ridge-like tissues (arrow) in 75% of the embryos (n = 8; scale bar: 200 μm). **(D) **Ectopic neural plate/ridge-like tissue (arrow) expresses high levels of the neural marker *Otx2 *(scale bar: 200 μm). For (C,D), dorsal view of the head region, anterior to the top. (E) Number of NSC colonies derived from 8.5-dpc anterior neural plate cells over-expressing *in vivo *the control construct alone (*EGFP*) or *EGFP *+ *Zfhx1b *(four separate experiments; n = 3 embryos (expressing control reporter construct) and n = 5 embryos (expressing *EGFP *+ *Zfhx1b *construct); two replicates per culture condition per treatment). The asterisk denotes a statistically significant result (*P *< 0.05) compared to the transfection control. S.d., standard deviation.

To further substantiate this model, we used microelectroporation to locally express *Zfhx1b *in the anterior ectoderm [35,36] while simultaneously blocking FGF signaling using SU5402 (Figure 6A). We hypothesized that if *Zfhx1b *was a downstream effector of the FGF-mediated cell fate transition, then its ectopic expression at a time when extremely few definitive NSCs are present (Figure 2A), and in the absence of FGF signaling, should facilitate the precocious transition of pNSCs to NSCs in the ectoderm itself. Electroporated embryos at approximately 7.0 dpc were cultured overnight and only those surviving embryos that demonstrated GFP reporter expression in the presumptive anterior neural plate were selected for further analysis (Figure 6B). Therefore, our procedure was adequate to facilitate the prospective dissection of regions of the anterior neural plate containing transfected cells in a selected population of embryos.

Over-expression of *Zfhx1b *was sufficient to induce an ectopic anterior neural plate/ridge-like structure in approximately 61% of the embryos and in the vast majority of cases it was unilateral (Additional file 4E). In one case the result was a greatly expanded anterior neural plate/ridge-like structure unilaterally (Figure 6C, arrow), but in most other instances a small unilateral anterior neural ridge-like tissue formed at the sight of over-expression. The cells in this expanded region demonstrated strong *Otx2 *gene expression (Figure 6D, arrow), suggesting they maintain their anterior neural identity within the anterior neural plate. Although most of the affected embryos demonstrated unilateral tissue expansion, in one instance we obtained morphological evidence of bilateral expansion of the anterior neural plate (Additional file 4C,D). These findings are consistent with the observation of an induced ectopic neural tissue in *Xenopus* ectoderm by *XSIP1* over-expression [27,28]. However, more detailed experiments are required to examine the extent of the morphological differentiation of these expanded neural plate/ridge-like structures. Importantly, we show that the early over-expression of *Zfhx1b *in ectoderm cells results in an approximately ten-fold increase in the number of FGF-responsive NSCs subsequently present in the 8.5-dpc anterior neural plate, despite the presence of the FGF receptor inhibitor (Figure 6E). The simultaneous derivation of LIF-responsive colonies in this experiment suggests that only those cells that were transfected were able to make the transition, while the many un-transfected cells remained as pNSCs (due to the presence of SU5402), suggesting that the effect is cell autonomous. Thus, *Zfhx1b *is sufficient to facilitate the precocious transition to a NSC identity in the absence of FGF signaling.

### Ectopic *Zfhx1b *expression is not sufficient to enhance definitive NSC proliferation and colony formation

A possible caveat to the interpretation presented thus far is that FGF signaling is a potent mitogen for NSCs. Therefore, an alternative interpretation is that the transition from pNSC to NSC *in vivo *may be independent of FGF-*Zfhx1b *signaling, but that the presence or absence of FGF-*Zfhx1b *signaling in the neurectoderm can modulate the size of the definitive NSC population via proliferation after the cell fate transition has occurred. Distinguishing between direct effects on cell fate versus selective proliferation can be difficult in this experimental context. However, one reason to suspect that the FGF-*Zfhx1b *signaling effects are not primarily mediated by selective proliferation is because in the co-injection experiments of DKK1 and SU5402 (Figure 4B), the results show an enhancement of FGF-responsive NSCs in the anterior neural plate compared to control injections despite the pre-exposure of the strong anti-mitogenic effects of the FGF receptor inhibitor SU5402.

**Figure 7:**
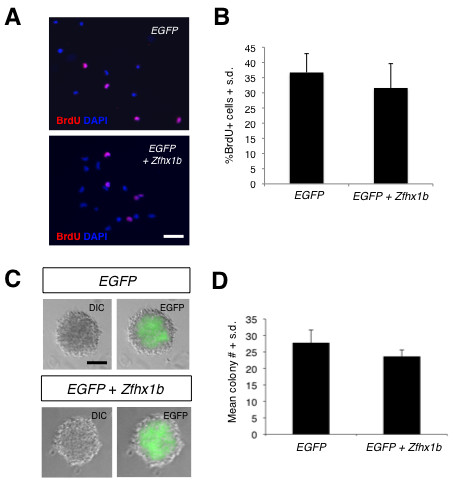
***Zfhx1b *over-expression is not sufficient to alter proliferation of definitive NSCs derived from the 14.0-dpc striatal germinal zone**. **(A) **Images of control (*EGFP*) transfected or *Zfhx1b + EGFP *transfected 14.5-dpc striatal germinal zone cells incorporating BrdU (red) with DAPI (blue) nuclear labeling (scale bar: 50 μm). **(B) **The percentage of BrdU+ cells in the control or *EGFP *+ *Zfhx1b *transfected cultures was not significantly different (*P *= 0.35); cultures plated in duplicate from n = 4 separate tissue samples in each group. **(C) **DIC and EGFP images of NSC colonies derived from 14.5-dpc striatal germinal zone cultures transfected with either control (*EGFP*) or *Zfhx1b *+ *EGFP *constructs (scale bar: 50 μm). DIC, differential interference contrast. **(D) **The number of FGF + H derived colonies from either the control or *Zfhx1b *transfected cultures was not significantly different (*P *= 0.51). The results represent the average from two separate experiments; n = 3 embryos per treatment group and four replicates per culture condition in each experiment. S.d., standard deviation.

Nonetheless, we sought to further determine whether FGF-*Zfhx1b *signaling could influence definitive NSC proliferation. We hypothesized that if *Zfhx1b *could enhance definitive NSC proliferation, then over-expression of *Zfhx1b *in NSCs derived from the 14.0-dpc striatal (that is, lateral ganglionic eminence) germinal zone should lead to an increase in proliferation in general, or an increase in the number and/or size of primary FGF-responsive NSC colonies. Freshly dissected striatal germinal zone cells were transiently transfected with *Zfhx1b *and *EGFP *constructs and cultured for either short-term bromodeoxyuridine (BrdU) incorporation experiments or in the NSC colony-forming assay. For short-term labeling 24 hours post-transfection, relatively high cell density cultures were pulsed for 3 hours with 10 μM BrdU and immediately analyzed by immunocytochemistry. There were 36% BrdU+ cells in the control transfected cultures compared to 31% BrdU+ cells in cultures transfected with *Zfhx1b *(Figure 7A, B). These data indicate that over-expression of *Zfhx1b *does not significantly enhance proliferation of neural precursor cells. For the colony forming assay, transfected cells were cultured at low cell densities for approximately 2 weeks, at which time the majority of clonally derived colonies contained some cells expressing GFP in both the control and *Zfhx1b *transfected cultures (Figure 7C). However, neither the number of colonies (Figure 7D) nor the size of the colonies (Additional file 5) was significantly different in the *Zfhx1b *transfected NSC cultures compared to controls. These data suggest that over-expression of *Zfhx1b *is not sufficient to alter the FGF-dependent proliferation of definitive NSCs. Thus, while we cannot eliminate the possibility of a differential effect on proliferation *in vivo *with absolute certainty, our data are consistent with the notion that acute modulation of FGF and Wnt signaling alters *Zfhx1b *expression in the neurectoderm to promote the cell fate transition from a pNSC identity to a NSC identity.

## Discussion

The results of several previous studies using ES cell models of mammalian neural induction found that FGF signaling, and downstream MAPK activation, was essential for the establishment of a definitive NSC identity *in vitro*, one that resembles the NSCs isolated from embryonic neural tube [17,20,22,37,38]. However, the ES cell model system also revealed that the initial specification of a primitive neural identity can occur independently of FGF signaling and was modulated primarily by the inhibition of well established repressors of vertebrate neural fate, such as BMP, Nodal and Wnt [15,17,20,39,40]. Importantly, others have observed in the ES cell model [37,41] and also in chick ectoderm [37] that FGF-dependent neural tissue formation is preceded by a primitive neurectodermal stage. Our present findings extend these previous results by showing that FGF signaling in the intact mouse ectoderm is required for definitive NSC identity to be established from a pNSC identity. We also demonstrate that the inhibition of Wnt signaling also facilitates the transition of a pNSC to a NSC identity. However, we do not yet know whether this pathway is strictly independent of FGF signaling (that is, without any cross-talk of the signal transduction cascades). Nonetheless, our data indicate that the activation of *Zfhx1b *gene expression is a common downstream effect of both FGF signaling and Wnt inhibition and is important for promoting the development of a definitive NSC identity in anterior ectoderm cells.

One question that remains to be clearly addressed is whether FGF signaling has an earlier role (that is, during or before gastrulation) in promoting the competence of mouse pluripotent epiblast cells to undergo neural commitment [6]. Indeed, others have demonstrated that the activation of Erk1/2 via autocrine FGF4 signaling in mouse ES cells may be necessary for lineage commitment (either neural or mesodermal) from a pluripotent state [22], and this is consistent with evidence for an early role of FGF in promoting the neural competence of ectoderm cells in *Xenopus *and chick (for example, [13,26]). Technical limitations (in particular embryo viability) prevented us from performing similar microinjection and electroporation experiments in the 6.0-dpc embryos to test this directly. However, we did observe that inhibition of FGF signaling at 7.0 dpc had no obvious effect on the LIF-dependent pNSC population at 7.5 dpc, suggesting that the main role for FGF was in the later transition to a definitive NSC identity. These data are congruent with the recent findings that FGF-induced Erk1/2 signaling controls the transition from an FGF5+ ectoderm identity (perhaps signifying a pNSC identity) to a definitive neural identity in mouse ES cells, and in its absence causes the maintenance or expansion of the ectoderm (presumably neural competent) state [37]. It is likely that the timing of FGF signaling [37,42] is critical at different stages of development and will be crucial in further elucidating its role in NSC fate specification in mouse.

Our study also reveals a novel role for *Zfhx1b *in mediating the transition to a definitive NSC identity. Indeed, ectopic expression of *Zfhx1b *in the absence of FGF signaling was sufficient to promote this lineage transition and as well cause ectopic neural plate tissue. This latter finding is consistent with evidence showing that vertebrate orthologs of *Zfhx1b/SIP1 *have a critical role in regulating neural induction in *Xenopus *[27,28], zebrafish [43,44], and chick [26]. In the case of chick neural induction, *Zfhx1b/SIP1 *function is downstream of FGF signaling and is mediated by the transcriptional activator *Churchill *[26]. Zebrafish *Churchill *has also been shown to regulate *sip1 *[45], suggesting that this relationship may be broadly conserved in vertebrates and worth investigating further in mouse, in particular with respect to NSC development.

*Zfhx1b/SIP1 *is known to interact with Smad proteins and mediate transcriptional repression of the TGFβ/BMP pathway [46-48]. This suggests that perhaps FGF-MAPK activation of *Zfhx1b *during NSC development acts to inhibit BMP-activated Smad signaling or represses *BMP4 *gene transcription, similar to what has been reported for FGF-MAPK signaling during *Xenopus *neural induction [49,50]. However, the Smad-binding domain is not required for the neural inductive capacity of *XSIP1 *[28]. Interestingly, mammalian *Zfhx1b *can function as a transcriptional activator and its repressor activity is regulated by post-translational modification [51]. Furthermore, a regulatory neural induction module of the *Xenopus Zic1 *gene contains *Zfhx1b *binding sites [52], as does the mouse *Sox1 *upstream regulatory domain (L Dang, V Tropepe, unpublished observations), suggesting that this protein may also function by directly activating transcription of genes that may be required for neural induction, such as *Zic1*. Consistent with this notion, recent evidence demonstrates that in the absence of BMP signaling, FGF provides an instructive signal that is necessary for activating early neural fate regulators, *Zic1 *and *Zic3*, in the *Xenopus *ectoderm [53].

*Fgfr1 *and *Fgfr2 *are expressed in the mouse ectoderm [54] and the aforementioned targeted deletion experiments suggest that either of these receptors are likely candidates for mediating the FGF signal that promotes NSC development *in vivo*. However, it remains uncertain which FGF ligand might be the predominant ligand regulating this process. The expression patterns and targeted deletion phenotypes of *Fgf4 *and *Fgf8 *appear to indicate that these ligands have an important role in FGF signaling in the ectoderm. Furthermore, *Fgf5 *is expressed in the early ectoderm [55], whereas *Fgf2 *and *Fgf1 *expression occurs primarily after neural plate formation [56]. Our NSC colony forming experiments revealed that FGF8b was as effective as FGF2 at facilitating colony formation, while FGF4 was significantly less effective. Therefore, *Fgf8*, and possibly *Fgf5*, may be the predominant ligand regulating the pNSC to NSC transition *in vivo*. However, given the substantial cross-activation between various FGF ligands and receptors, further experiments are required to discern the precise role for specific FGF-FGF receptor interactions *in vivo*.

The orphan nuclear receptor germ cell nuclear factor (GCNF) also regulates the transition from a pNSC to a NSC by repressing *Oct4 *expression in the neurectoderm [57]. The onset of expression of *GCNF *seems to parallel that of *Zfhx1b*, and in the 8.5-dpc neural plate of *GCNF-/- *embryos, there is an increased number of LIF-responsive pNSCs that persist, along with a concomitant decrease in the number of definitive NSCs [57]. Thus, it is possible that *GCNF *expression may be modulated downstream of FGF signaling or in a parallel pathway. It will be interesting to determine the regulatory relationship between the *GCNF*, *Zfhx1b *and FGF signaling in order to provide further insight into the molecular mechanism of NSC development.

The mouse *Zfhx1b*/*SIP1 *knockout demonstrates severe neurodevelopmental malformations [25]. It was reported that the expression of the early neural gene *Sox2 *was reduced in the neural plate, likely due to the persistence of the expression of *E-cadherin*, which further compromised the development of the neural plate [25]. Given our findings that *Zfhx1b *is important for definitive NSC formation in the anterior neurectoderm, we speculate that there is a significant defect in NSC development within the neural plate of the *Zfhx1b *knockout mouse. Mutations or deletions in the human *ZFHX1B *gene are strongly associated with Mowat-Wilson syndrome (OMIM 235730), a severe form of mental retardation [58], suggesting that part of the phenotypic spectrum of this human syndrome may involve a defect in early NSC development.

## Conclusions

Our study implicates FGF signaling, and the inhibition of Wnt signaling, in the activation of *Zfhx1b *gene expression in the mouse anterior ectoderm and in promoting the transition of a pNSC to a definitive NSC cell identity during neural plate formation. The mouse *Zfhx1b *gene is critical for early neurodevelopment and mutations in the human ortholog have been strongly implicated in causing Mowat-Wilson syndrome, a form a mental retardation. Therefore, our study provides a new perspective on studying the etiology of neurodevelopmental disorders by implicating possible defects in NSC development as a contributing factor underlying disease pathogenesis.

## Methods

### Mouse whole embryo microinjection, electroporation and culture

CD1 mice (Charles River Laboratories, St Constant, Quebec, Canada) were treated in accordance with the regulations on animal experimentation established by the Canadian Council on Animal Care. The experimental procedures were approved by the University of Toronto Animal Care Committee. Embryos from timed-pregnant mice were obtained by dissection from their decidua in 1:1 DMEM:PBS. Reichert's membrane was completely removed using tungsten needle and forceps. Embryos were microinjected using 1.0 mm glass capillary needles (World Precision Instruments, Sarasota, Florida, USA) with the IM 300 Microinjector and subsequently cultured *ex vivo *in 1:1 DMEM:FBS (Gibco/Stem Cell Technologies, Vancouver, British Columbia, Canada) at 37°C, 5% CO_2 _for either 4 hours or 24 hours. Recombinant proteins FGF8b (20 ng/ml), FGF2 (20 ng/ml), Wnt3a (100 ng/ml), DKK1 (100 ng/ml), and FGF4 (25 ng/ml) (R&D Systems, Minneapolis, Minnesota, USA) were used according to the manufacturers' instructions. MEK inhibitor, U0126 (Calbiochem-EMD, Gibbstown, New Jersey, USA; gift from Winklbauer Lab) was used at 20 μM in 0.1% DMSO, and FGF receptor inhibitor, SU5402 (Calbiochem; gift from Bonfanti Lab) was used at 5 μM in 0.05% DMSO. 1× PBS as well as 0.05% or 0.1% DMSO was used as injection controls. For the NSC colony-forming assay, standard protocols were used [17,24] and cells were plated at five cells per microliter in 96-well plates and colonies were counted 14 days after plating. For mouse embryo electroporation [35,36], midstreak embryos were dissected and injected as described. Plasmids (*Zfhx1b/SIP1*, NM_015753, OriGene, Rockville, Maryland, USA) or *EGFP *(from Tam Lab) were injected at 6 μg/μl along with SU5402 (5 μM) in 0.05% DMSO into the amniotic cavity and incubated at room temperature in PBS for at least 5 minutes prior to electroporation. Embryos were positioned between a positive plate electrode (flattened platinum wire) and a negative point electrode (sharpened tungsten wire) with the anterior of the embryo oriented towards the positive plate electrode and were held in place by a holding pipette (hand pulled, cut and microforged), suspended in Tyrode Ringer's Saline pH 7.5. Electroporation was carried out using the BTX Electro Square Porator ECM830 (Harvard Apparatus, Holliston, Massachusetts, USA) at 15V, Path length 0050.6 ms, low voltage mode, 20 ms/pulse, 5 pulses total. Embryos were cultured as described for up to 24 hours. Schematics of mouse embryo sections were adapted from emap: the Edinburgh Mouse Atlas Project [59].

### Cell transfection

Transfection was performed using Lipofectamine 2000 (Invitrogen, Carlsbad, California, USA) using 1 μg/well DNA concentrations in 96-well plates. Fluorescence of cells was visualized 24 hours following transfection and colonies derived from tranfected cultures were counted 14 days after plating. The full-length *Zfhx1b*/*SIP1 *construct was obtained through Origene (NM_015753) and the *EGFP *over-expression construct was a gift from Tam Lab.

### Neural stem cell colony forming (neurosphere) assay

Anterior neurectoderm (7.5 dpc), anterior neural plate (8.5 dpc) and lateral ganglionic eminence (14.5 dpc) dissections and NSC cultures (cells plated at 5 to 10 cells/μl in the presence of FGF2 and supplemented with heparin were performed as previously described [23,24]. Means and standard deviations were calculated for the control and experimental treatment groups and statistical differences between groups were determined using a Student's *t*-test.

### Western blot

Embryos were injected and cultured for 24 hours as described and tissue samples were collected in ice cold lysis buffer containing 10 mM Tris-Cl pH 7.5, 50 mM NaCl, 1 mM EDTA, 1 mM phenylmethanesulfonylfluoride, and Halt phosphatase inhibitor (Thermo Fisher Scientific, Waltham, Massachusetts, USA). The supernatant was subsequently used at 1:1 dilution in protein sample buffer (100 mM Tris-Cl pH 6.8, 30 mM β-mercaptoethanol, 4% SDS, 20% glycerol, 0.01% bromophenol blue). Samples were run on a 10% SDS-PAGE gel and blotted (methanol based transfer) for 2 hours, 30 V constant. Western blotting was performed for Zfhx1b/SIP1 (rabbit anti-SIP1 antibody, 1:1,000; Abcam, Cambridge, Massachusetts, USA), pERK1/2 (rabbit anti-pERK1/2, 1:1,000; Cell Signaling, Danvers, Massachusetts, USA), ERK1/2 (rabbit anti-ERK1/2, 1:1,000; Cell Signaling), and tubulin (mouse anti-Tubulin, 1:1,000; Cell Signaling) following the suggested protocol. Secondary goat anti-rabbit or rabbit anti-goat antibodies conjugated with horse radish peroxidase (Jackson Immunoresearch, West Grove, Pennsylvania, USA) were used at 1:1,000. ECL analysis was used to visualize bands using the ECL Detection Kit (GE Health Care, Piscataway, New Jersey, USA) following the standard protocol.

### *In situ *hybridization

Plasmids containing *Zfhx1b*/*SIP1 *(gift from Tylzanowski Lab) and *Otx2 *(gift from Rossant Lab) were used to generate antisense RNA probes using digoxigenin-11-uridine 5'-triphosphate (Roche, Laval, Quebec, Canada) following the standard protocol. Embryos were fixed in 4% paraformaldehyde at stages indicated, and whole mount *in situ *hybridization was performed. Digoxigenin-labeled probes were detected with anti-DIG alkaline phosphatase-coupled Fab fragment (1:2,000; Roche). Alkaline phosphatase reaction was performed using BM Purple (Roche).

### Quantitative and semi-quantitative PCR

For semi-quantitative PCR, total RNA was extracted with the mini to midi RNA extraction kit (Invitrogen). First strand cDNAs were reverse transcribed from oligo(dT)_12-18_-primed total RNA (DNase treated, 0.5 μg) using Super-Script III (Invitrogen). Each 25 μl reaction consisted of 1.6× PCR buffer, 1.5 mM MgCl_2_, 0.2 mM deoxyribonucleotide triphosphates (dNTPs), 0.4 μM each of forward and reverse primers, 0.5 U Platinum Taq DNA polymerase, and diluted cDNA template (1:50). PCR conditions were as follows: 95°C for 3 minutes, 27 to 35 cycles of 95°C for 30 s, 54 to 59°C for 30 s, 72°C for 45 to 60 s. Annealing temperatures and cycle number for each primer pair (Additional file 6) were determined using gradient PCR (MJ Research, Waltham, Massachusetts, USA). Products were resolved on a 1% agarose gel. For real-time RT-PCR the Rotor-Gene 3000 Thermal Cycler (Corbett Research, Cambridge, United Kingdom) was used. First-strand cDNAs were reversed transcribed as described. Each 25 μl reaction consisted of 1.6× PCR buffer, 3 mM MgCl_2_, 0.2 mM dNTPs, 0.2 μM each of forward and reverse primers (Additional file 6), 0.5 U Platinum Taq DNA polymerase, 1.0× SYBR Green I (Invitrogen), and diluted cDNA template (1:50). Test samples were carried out in duplicate, including a control reaction with template omitted: 95°C for 10 minutes, 50 cycles of 95°C for 15 s, 55°C for 30 s, and 72°C for 45 s. Post-PCR amplification for melt curve analyses was performed by ramping from 72 to 99°C at 0.2°C/s to check the specificity of the amplicons. PCR products were verified on a 1% agarose gel to ensure correct amplification sizes were obtained. Quantification of relative gene expression (using *β-actin *gene expression as a control) was calculated with Rotor-Gene Software, version 6.0 (Corbett Research).

## Abbreviations

BMP: bone morphogenetic protein; BrdU: bromodeoxyuridine; DMEM: Dulbecco's modified Eagle's medium; dpc: days post-coitum; EGFP: enhanced green fluorescent protein; ES: embryonic stem; FBS: fetal bovine serum; FGF: fibroblast growth factor; GCNF: germ cell nuclear factor; GFP: green fluorescent protein; LIF: leukemia inhibitory factor; MAPK: mitogen-activated protein kinase; NSC: neural stem cell; PBS: phosphate-buffered saline; pNSC: primitive neural stem cell; TGF: transforming growth factor.

## Competing interests

The authors declare that they have no competing interests.

## Authors' contributions

LD participated in designing experiments, performed almost all of the experiments, participated in data analysis and interpretation, and helped to draft and edit the manuscript. VT conceived of the study and participated in its design, coordination, performing experiments, data analysis and interpretation, and drafted the manuscript. All authors read and approved the final manuscript.

## Supplementary Material

Additional file 1 **Microinjection is more efficient at altering expression of neural specification genes at short time intervals, but does not alter broad anteroposterior patterning**. **(A) **Control experiment showing embryos injected (I) with PBS or Wnt3a versus cultured **(C) **with the addition of PBS or Wnt3a to culture media. Semi-quantitative RT-PCR shows that by microinjecting into the pro-amniotic cavity, we are capable of modulating gene expression more efficiently after 4 hours than by exposing the embryos to the same factors in culture for the same time. Tissue prepared as described in Figure 1. **(B) **Embryos were microinjected with either PBS or FGF8b and cultured *ex vivo *for approximately 24 hours. Tissue samples prepared as described in Figure 1, and semi-quantitative RT-PCR demonstrates that broad anteroposterior patterning of the cranial neural plate is not altered. Click here for file

Additional file 2 **Exposure to SU5402 does not acutely alter the proportion of LIF-dependent pNSCs and does not affect longer term FGF signaling**. **(A) **Embryos injected at 7.0 dpc with either DMSO or SU5402 and cultured *ex vivo *for approximately 4 hours. Anterior neural tissue was processed and cultured in the colony forming assay in the presence of LIF; n = 6 samples per injection group. The number of colonies generated was not significantly different between the two groups. **(B) **Embryos injected at 7.0 dpc with SU5402 (or DMSO) were cultured *ex vivo *for approximately 24 hours and the anterior neural plate tissue of the SU5402-treated group was thoroughly rinsed, dissected and resuspended in fresh serum-free media with FGF2 + heparin (H), incubated for 2 hours and then harvested for western blotting to assay for phospo-Erk1/2 (pErk1/2), Erk and Actin; n = 5 embryos per group. Level of pErk1/2 in SU5402 treated, FGF + H cultured tissue is similar to DMSO injected controls. Click here for file

Additional file 3 **Production of *Zfhx1b *mRNA and protein in transfected HEK293 cells**. **(A) **Transient transfection and over-expression of *Zfhx1b *in 293T cells. Cells were collected for RT-PCR after 24 hours following transfection. RT-PCR showing presence of mouse *Zfhx1b *mRNA, in human 293T *Zfhx1b* transfected samples, but not in untransfected control. Similarly, western blotting shows the presence of *Zfhx1b* protein. **(B)** Anterior neural plates at 8.5 dpc were dissected from embryos injected with either DMSO or SU5402 at 7.0 dpc, and grown for 24 hours in DMEM:FBS. Anterior neural plates were triturated, transfected with a control vector (*EGFP*) or the *Zfhx1b *over-expression construct and cultured in low-density serum-free media. Fluorescence can be visualized following 24 hours and the images (DIC or EGFP show representative live cells (top panels, left arrow, and bottom panels), as well as a non-labeled cell (top panels, right arrow). Scale bar: 10 μm. DIC, differential interference contrast. Click here for file

Additional file 4 **Expanded neural plate/ridge-like tissue in embryos electroporated with *Zfhx1b***. **(A) **Dorsal view of an electroporated embryo with no evidence for tissue expansion (similar to untreated controls). Anterior to the top. **(B) **Lateral view of the embryo in (A) highlighting the contours of the anterior neural plate (green line). Anterior to the right. **(C) **Dorsal view of an electroporated embryo with evidence for a bilateral expansion of the anterior neural plate (red dotted line). Anterior to the top. **(D) **Lateral view of the embryo in (C) highlighting the contours of the normal region of the anterior neural plate (green line) and the contour of the expanded neural plate/ridge-like tissue (red line). Anterior to the right. a, allantois; h, heart. **(E) **Table summarizing the results of the electroporation of *Zfhx1b *at 7.0 dpc. A total of n = 18 embryos from two separate experiments are represented. Click here for file

Additional file 5 **Zfhx1b does not alter the growth rate of FGF-dependent NSC colonies**. The average colony size (diameter in microns) of FGF + heparin (H) derived colonies from either the control or *EGFP *+ *Zfhx1b *transfected cultures was not significantly different (*P *= 0.41). The results represent the average from two separate experiments; n = 3 embryos per treatment group and 4 replicates per culture condition in each experiment. Click here for file

Additional file 6 **Semi-quantitative and quantitative PCR primer sequences and expected amplicon size**. Semi-quantitative and quantitative PCR primer sequences and expected amplicon size. Click here for file
