# Supplementary figures and images for: FGF dependent regulation of Zfhx1b gene expression promotes the formation of definitive neural stem cells in the mouse anterior neurectoderm
Source: Neural Dev. 2010 May 6;5:13. doi: 10.1186/1749-8104-5-13 (PMC2883982; doi:10.1186/1749-8104-5-13)

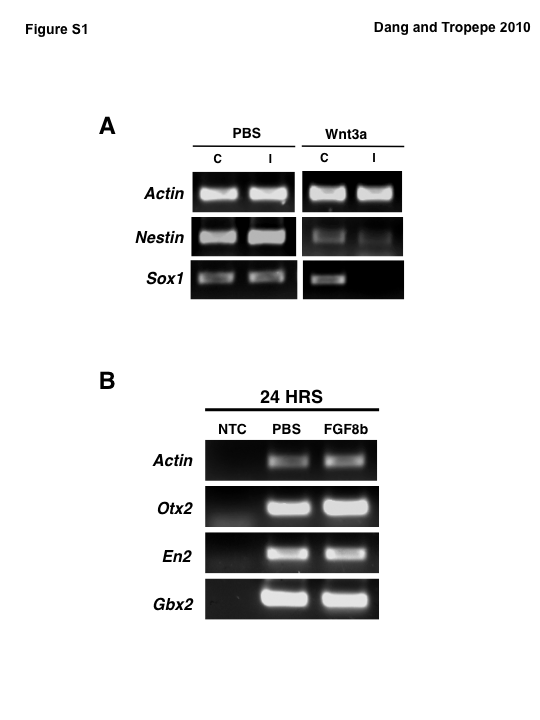

Supplement: Additional file 1 — Microinjection is more efficient at altering expression of neural specification genes at short time intervals, but does not alter broad anteroposterior patterning. (A) Control experiment showing embryos injected (I) with PBS or Wnt3a versus cultured (C) with the addition of PBS or Wnt3a to culture media. Semi-quantitative RT-PCR shows that by microinjecting into the pro-amniotic cavity, we are capable of modulating gene expression more efficiently after 4 hours than by exposing the embryos to the same factors in culture for the same time. Tissue prepared as described in Figure 1. (B) Embryos were microinjected with either PBS or FGF8b and cultured ex vivo for approximately 24 hours. Tissue samples prepared as described in Figure 1, and semi-quantitative RT-PCR demonstrates that broad anteroposterior patterning of the cranial neural plate is not altered. [file 1749-8104-5-13-S1.TIFF]

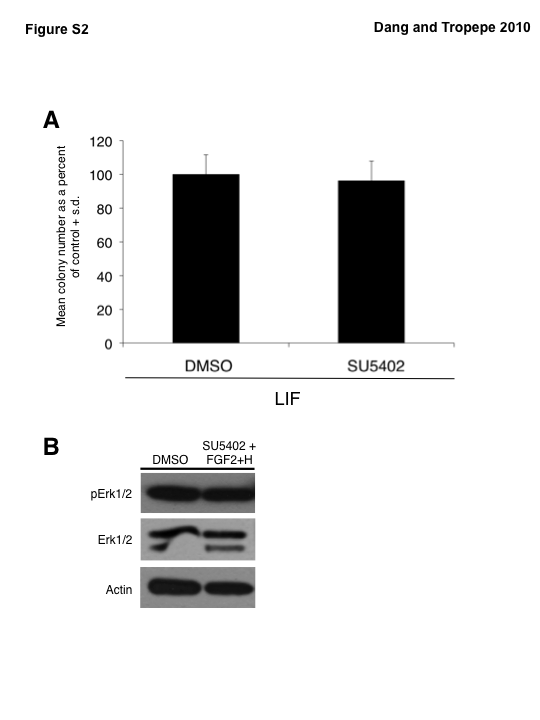

Supplement: Additional file 2 — Exposure to SU5402 does not acutely alter the proportion of LIF-dependent pNSCs and does not affect longer term FGF signaling. (A) Embryos injected at 7.0 dpc with either DMSO or SU5402 and cultured ex vivo for approximately 4 hours. Anterior neural tissue was processed and cultured in the colony forming assay in the presence of LIF; n = 6 samples per injection group. The number of colonies generated was not significantly different between the two groups. (B) Embryos injected at 7.0 dpc with SU5402 (or DMSO) were cultured ex vivo for approximately 24 hours and the anterior neural plate tissue of the SU5402-treated group was thoroughly rinsed, dissected and resuspended in fresh serum-free media with FGF2 + heparin (H), incubated for 2 hours and then harvested for western blotting to assay for phospo-Erk1/2 (pErk1/2), Erk and Actin; n = 5 embryos per group. Level of pErk1/2 in SU5402 treated, FGF + H cultured tissue is similar to DMSO injected controls. [file 1749-8104-5-13-S2.TIFF]

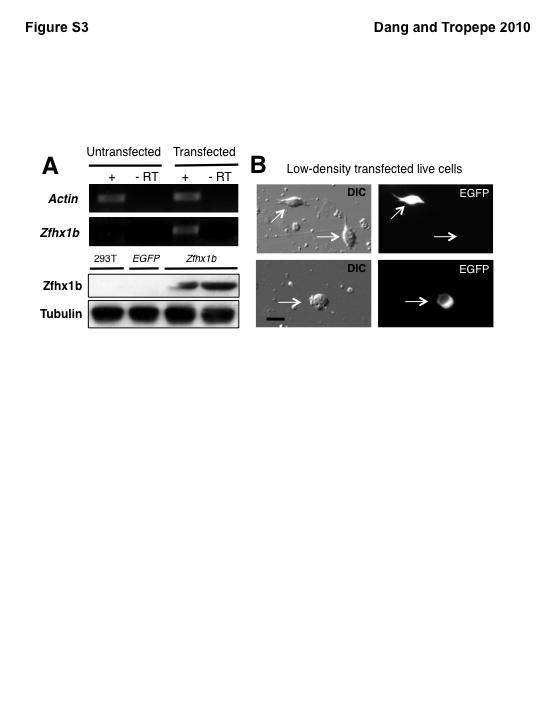

Supplement: Additional file 3 — Production of Zfhx1b mRNA and protein in transfected HEK293 cells. (A) Transient transfection and over-expression of Zfhx1b in 293T cells. Cells were collected for RT-PCR after 24 hours following transfection. RT-PCR showing presence of mouse Zfhx1b mRNA, in human 293T Zfhx1b transfected samples, but not in untransfected control. Similarly, western blotting shows the presence of Zfhx1b protein. (B) Anterior neural plates at 8.5 dpc were dissected from embryos injected with either DMSO or SU5402 at 7.0 dpc, and grown for 24 hours in DMEM:FBS. Anterior neural plates were triturated, transfected with a control vector (EGFP) or the Zfhx1b over-expression construct and cultured in low-density serum-free media. Fluorescence can be visualized following 24 hours and the images (DIC or EGFP show representative live cells (top panels, left arrow, and bottom panels), as well as a non-labeled cell (top panels, right arrow). Scale bar: 10 μm. DIC, differential interference contrast. [file 1749-8104-5-13-S3.TIFF]

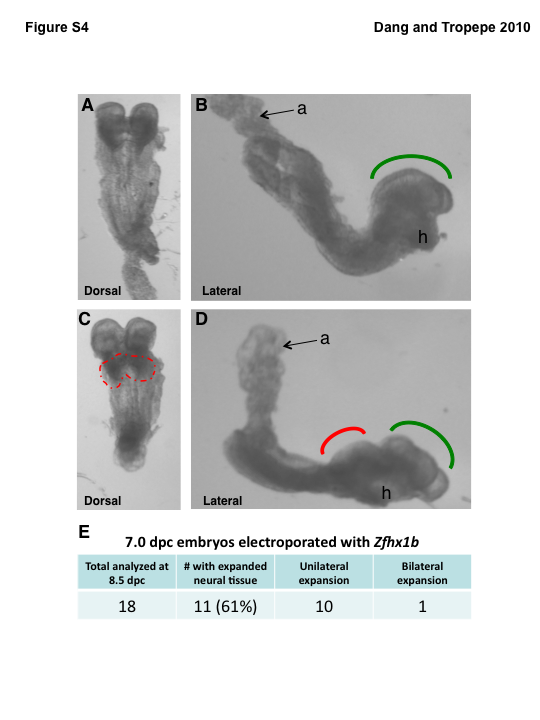

Supplement: Additional file 4 — Expanded neural plate/ridge-like tissue in embryos electroporated with Zfhx1b. (A) Dorsal view of an electroporated embryo with no evidence for tissue expansion (similar to untreated controls). Anterior to the top. (B) Lateral view of the embryo in (A) highlighting the contours of the anterior neural plate (green line). Anterior to the right. (C) Dorsal view of an electroporated embryo with evidence for a bilateral expansion of the anterior neural plate (red dotted line). Anterior to the top. (D) Lateral view of the embryo in (C) highlighting the contours of the normal region of the anterior neural plate (green line) and the contour of the expanded neural plate/ridge-like tissue (red line). Anterior to the right. a, allantois; h, heart. (E) Table summarizing the results of the electroporation of Zfhx1b at 7.0 dpc. A total of n = 18 embryos from two separate experiments are represented. [file 1749-8104-5-13-S4.TIFF]

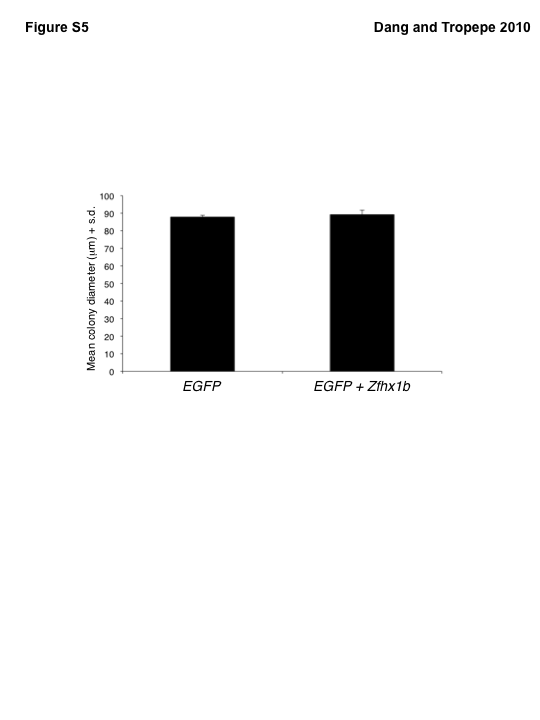

Supplement: Additional file 5 — Zfhx1b does not alter the growth rate of FGF-dependent NSC colonies. The average colony size (diameter in microns) of FGF + heparin (H) derived colonies from either the control or EGFP + Zfhx1b transfected cultures was not significantly different (P = 0.41). The results represent the average from two separate experiments; n = 3 embryos per treatment group and 4 replicates per culture condition in each experiment. [file 1749-8104-5-13-S5.TIFF]
