# Supplementary material for: FGF dependent regulation of Zfhx1b gene expression promotes the formation of definitive neural stem cells in the mouse anterior neurectoderm
Source: Neural Dev. 2010 May 6;5:13. doi: 10.1186/1749-8104-5-13 (PMC2883982; doi:10.1186/1749-8104-5-13)
Supplement: Additional file 6 — Semi-quantitative and quantitative PCR primer sequences and expected amplicon size. Semi-quantitative and quantitative PCR primer sequences and expected amplicon size. [file 1749-8104-5-13-S6.DOC]

Semi-quantitative PCR primer sequences and expected amplicon size

| **Gene** | **Forward Primer** | **Reverse Primer** | **Amplicon** |
| --- | --- | --- | --- |
| *β-actin* | TGTTACCAACTGGGACGACA | TCTCAGCTGTGGTGGTGAAG | 392 bp |
| *Oct4* | TCAGACTTCGCCTCCTCAC | TCCAGACTCCACCTCACAC | 679 bp |
| *Nanog* | GCTTACAAGGGTCTGCTAC | AATGGATGCTGGGATACTC | 413 bp |
| *Sox2* | GACTGCGAACTGGAGAAG | GAAAGGGTAGGATTGAAC | 660 bp |
| *Nestin* | CAACCCTCACCACTCTAT | CCTCTGCTTCTTCAAATC | 731 bp |
| *Zfhx1b* | CGTGGTGAACTATGACAAC | GCTACAAAGAGGGCAGGAA | 651 bp |
| *Zfhx1a* | CACCGCCGTCATTTATCC | CACTCACTGCTTCTTGCTC | 916 bp |
| *T* | CCGGTGCTGAAGGTAAATGT | TGACCGGTGGTTCCTTAGAG | 317 bp |
| *Gsc* | GAAGCCCTGGAGAACCTCTT | CCGAGTCCAAATCGCTTTTA | 593 bp |
| *Hesx1* | ACAGACCCTGGACAGACACC | TTCGTCCTCGGTACCAACTC | 699 bp |
| *Otx2* | CCATGACCTATACTCAGGCTTCAGG | GAAGCTCCATATCCCTGGGTGGAAAG | 211 bp |
| *En2* | AGCTCAGCCTGAACGAGTCT | CATTGTTTCTCGCGGCCCTA | 222 bp |
| *Gbx2* | CGGCAACTTCGACAAAGC | AGAGAGAAGCTCTCCTCCTTGC | 198 bp |

| **Gene** | **Forward Primer** | **Reverse Primer** | **Amplicon** |
| --- | --- | --- | --- |
| *β-actin* | TGTTACCAACTGGGACGACA | TCTCAGCTGTGGTGGTGAAG | 392 bp |
| *Zfhx1b* | CTATTCCCCTGCATCAGCAT | GGCTTGTCAGTCCTTTCTCG | 165 bp |

Quantitative PCR primer sequences and expected amplicon size
